# A Zinc-Dependent Protease AMZ-tk from a Thermophilic Archaeon is a New Member of the Archaemetzincin Protein Family

**DOI:** 10.3389/fmicb.2015.01380

**Published:** 2015-12-17

**Authors:** Baolei Jia, Zhengqun Li, Jinliang Liu, Ying Sun, Xiaomeng Jia, Yuan Hu Xuan, Jiayan Zhang, Che Ok Jeon

**Affiliations:** ^1^Department of Life Science, Chung-Ang UniversitySeoul, South Korea; ^2^College of Plant Sciences, Jilin UniversityChangchun, China; ^3^College of Plant Protection, Shenyang Agricultural UniversityShenyang, China

**Keywords:** zinc-dependent protease, archaemetzincin, thermophilic archaeon, enzymatic property, biotechnological applications

## Abstract

A putative zinc-dependent protease (TK0512) in *Thermococcus kodakarensis* KOD1 shares a conserved motif with archaemetzincins, which are metalloproteases found in archaea, bacteria, and eukarya. Phylogenetic and sequence analyses showed that TK0512 and its homologues in *Thermococcaceae* represent new members in the archaemetzincins family, which we named AMZ-tk. We further confirmed its proteolytic activity biochemically by overexpression of the recombinant AMZ-tk in *Escherichia coli* and characterization of the purified enzyme. In the presence of zinc, the purified enzyme degraded casein, while adding EDTA strongly inhibited the enzyme activity. AMZ-tk also exhibited self-cleavage activity that required Zn^2+^. These results demonstrated that AMZ-tk is a zinc-dependent protease within the archaemetzincin family. The enzyme displayed activity at alkaline pHs ranging from 7.0 to 10.0, with the optimal pH being 8.0. The optimum temperature for the catalytic activity of AMZ-tk was 55°C. Quantitative reverse transcription-PCR revealed that transcription of *AMZ-tk* was also up-regulated after exposing the cells to 55 and 65°C. Mutant analysis suggested that Zn^2+^ binding histidine and catalytic glutamate play key roles in proteolysis. AMZ-tk was thermostable on incubation for 4 h at 70°C in the presence of EDTA. AMZ-tk also retained >50% of its original activity in the presence of both laboratory surfactants and commercial laundry detergents. AMZ-tk further showed antibacterial activity against several bacteria. Therefore, AMZ-tk is of considerable interest for many purposes in view of its activity at alkaline pH, detergents, and thermostability.

## Introduction

Most organisms typically employ 2–4% of their genetic resources to code for proteases (EC 3.4.11-19/21-25/99), which are one of the most abundant classes of enzymes and are involved in a wide range of biological processes, including cell-cycle progression, cell signaling, proliferation and death, protein trafficking and immune response (Deu et al., 2012; Hu et al., 2012). Proteases differ in properties such as substrate specificity, active site and catalytic mechanism. Proteases are broadly classified as endo- or exo-enzymes on the basis of their site of action on protein substrates, and they are further categorized as serine proteases, aspartic proteases, cysteine proteases or metalloproteases based on the residue or cofactor essential for catalysis (Rao et al., 1998). Metalloproteases contain a metal ion at their active site that acts as a catalyst in the hydrolysis of peptide bonds. Most metalloproteases require zinc (Zn^2+^), but other transition metals have been found at active sites, such as cobalt (Co^2+^) and manganese (Mn^2+^) (Holmquist and Vallee, 1974; Häse and Finkelstein, 1993). Generally, metal ions are bound in a nearly tetrahedral conformation via three ligands that vary with histidine, glutamate, aspartate, lysine, and arginine. The fourth coordination position is taken up by a labile water molecule (Vallee and Auld, 1990). Among the metal proteases, archaemetzincin or archaelysin (MEROPS family M54.001) is a zinc-dependent aminopeptidase that contains the consensus zinc-binding sequence HExxHxxGx_3_Cx_4_CxMx_17_CxxC and a conserved Met residue at the active site. Archaemetzincins, first identified in archaea, are also found in vertebrates, including birds, amphibians, and fish. There are two human members, archaemetzincin-1 and -2 (AMZ1 and AMZ2). AMZ1 is mainly found in the liver and heart while AMZ2 is primarily expressed in the testis and heart; both have been reported to degrade synthetic substrates and peptides (Diaz-Perales et al., 2005). Previous bioinformatics analysis revealed no orthologous sequences in plants (Diaz-Perales et al., 2005). However, as more genome sequences have become available, archaemetzincins have been identified in plants; for example, *Pyrus bretschneideri* (XP_009350639.1), which was annotated as an uncharacterized protein (Wu et al., 2013).

Proteases are one of the most important groups of industrial enzymes. They are one of the three largest groups of industrial enzymes, accounting for approximately 60% of the total worldwide sales of enzymes (Niehaus et al., 1999). Proteases have a large variety of applications, mainly in the food processing, feed, metal recovery, detergent, leather processing, waste treatment, and chemical industries, as well as for medical uses. Proteases are also used in basic research. For example, their property of selective cleavage of peptide bonds is used in the elucidation of structure-function relationships (Rao et al., 1998).

Over the last decade, *Thermococcus kodakarensis* KOD1, a potential industrial enzyme source, has been developed into a model system for studying archaeal molecular mechanisms, leading to major research progress in studying sugar metabolism (Guan et al., 2013; Han et al., 2013), amino acid synthesis (Rahman et al., 1998; Hiyama et al., 2014), gene transcription (Jager et al., 2014), and other processes (Jia et al., 2015). Although proteases are important players in cellular life, only a subtilisin homolog from *T. kodakarensis*, KOD1 (Tk-SP), has been studied intensively. Tk-SP maintains its proteolytic activity in the presence of non-ionic surfactants and EDTA at 80°C (Foophow et al., 2010; Hirata et al., 2013). Tk-SP degrades the abnormal prion protein, which is highly resistant to normal sterilization procedures and proteolytic enzymes (Koga et al., 2014). Most proteases predicted for *T. kodakarensis* KOD1 remain to be characterized both for their biochemical properties and their physiological functions. In this report, we cloned and overexpressed a zinc-dependent protease (Tk0512, annotated as peptidase M54), which is a homolog of archaemetzincin, from *T. kodakarensis* (named AMZ-tk). We also biochemically characterized the enzyme and examined its enzymatic properties.

## Materials and Methods

### Microorganisms and Media

*Thermococcus kodakarensis* KOD1, which was kindly donated by the Japan Collection of Microorganisms, RIKEN BioResource Center, Japan, was used to isolate genomic DNA, and it was cultured in 280 *Thermococcus* medium (Fukui et al., 2005).

### Cloning of AMZ-tk

PCR using *T. kodakarensis* KOD1 genomic DNA as a template was performed to isolate *AMZ-tk* using the following oligonucleotide primers: forward: 5′-CCG GAATTC ATG GAG TAC ATA GGC TTC ACA TAC G-3′ and reverse: 5′-CCG CTCGAG TTA ATC TTT CCC CTT GGT ATC TGC-3′ (the underlined bases indicate the restriction enzyme sites for *Eco*RI and *Xho*I). The PCR product and the pET28-(a) vector were digested by the restriction enzymes and ligated. The construct with N-terminal His-tag was transformed into *Escherichia coli* BL21 (DE3) cells by electroporation and confirmed by sequencing.

### Expression and Purification of AMZ-tk

*Escherichia coli* BL21 (DE3) cells containing the pET28a-AMZ-tk plasmid were cultured in 2 L of LB broth containing kanamycin (30 μg.mL^-1^) at 37°C for 3 h. When the OD_600_ reached 0.7, isopropyl-β-D-thiogalactopyranoside (IPTG) was added to a final concentration of 1 mM to induce protein expression. After 4 h of culture with shaking, cells were harvested by centrifugation at 6,000 rpm for 10 min at 4°C. The cell pellets were resuspended in lysis buffer containing 50 mM Tris-HCl (pH 8.0), 300 mM NaCl, 20 mM 2-mercaptoethanol, and 20 mM imidazole. The cell suspension was sonicated and heated at 65°C for 1 h. The thermostable components in the supernatant were collected following centrifugation and loaded on a Ni-NTA column. After washing the column with lysis buffer, AMZ-tk was eluted using an imidazole gradient (50–250 mM). The purified AMZ-tk was visualized after separation by 12% sodium dodecyl sulphate polyacrylamide gel electrophoresis (SDS-PAGE). After dialysis with 50 mM Tris-HCl buffer (pH 8.0) containing 300 mM NaCl and 20 mM 2-mercaptoethanol to remove any metal ion, the purified proteins were stored in -80°C. Protein concentrations were estimated by the method of Bradford using bovine serum albumin (BSA) as a standard (Bradford, 1976).

The primers used for the single amino acid mutant were as follows: H182D, forward primer, 5′-GGC ATG CTC GAC GAG ATT GGG; reverse primer, 5′- CCC AAT CTC GTC GAG CAT GCC; H186D, forward primer, 5′-GAG ATT GGG GAC TCT TTC GGG; reverse primer, 5′-CCC GAA AGA GTC CCC AAT CTC; H192D, forward primer, 5′-GGG CTG GAA GAC TGT CCC AAC, reverse primer, 5′-GTT GGG ACA GTC TTC CAG CCC; E183Q, forward primer, 5′-ATG CTC CAC CAA ATT GGG CAC; reverse primer, 5′-GTG CCC AAT TTG GTG GAG CAT. The PCR was performed using Pfu polymerase, and the cycling parameters were: 95°C for 5 min (one cycle), 95°C for 30 s, and 68°C for 12 min (12 cycles). After amplification, the PCR mixture was digested with *Dpn*I and then transformed into *E. coli* BL21(DE3) by electroporation. The mutants were confirmed by DNA sequencing. The mutant proteins were purified using the same method as used for wild-type purification.

### Assays for Protease Activity

The proteolytic activity of the purified proteins was determined using casein as substrate. A reaction volume of 500 μL was prepared in a microfuge tube containing purified AMZ-tk (25 μg) and casein (325 μg) dissolved in 50 mM Tris-HCl (pH 8.0) containing 300 mM NaCl, and 0.8 mM Zn^2+^ or indicated amount of other metal ions. After incubation at 55°C for 30 min, the reaction was stopped by adding 50 μL of 50% trichloroacetic acid to precipitate unreacted substrates. After removing the precipitates by centrifugation (12,000 *g*, 10 min), the enzymatic products in the supernatant were quantified at 280 nm using a UV-visible spectrophotometer (Shimadzu, Japan). The specific activity of the enzyme (units.mg^-1^.min^-1^) was expressed as the amount of enzyme required to produce 1 μM of tyrosine under the assay conditions.

The influence of pH on AMZ-tk activity was determined using the protocol described above with the exception of replacing the Tris-HCl buffer with 50 mM sodium acetate (pH 3.0–5.0), 50 mM MES (pH 5.0–7.5), 50 mM HEPES (pH 8.0–8.5), or 50 mM glycine (pH 9.0–10.0). All assays were performed at the optimal temperature. To determine the influence of temperature on the enzymatic activity, samples were incubated at temperatures from 30 to 80°C for 30 min. Thermostability was investigated after incubation of the samples at different temperatures at optimal pH. After various time intervals, samples were withdrawn, and the enzymatic activity was measured.

The effects of various metal ions, EDTA, and other chemicals on the hydrolytic activity of AMZ-tk were determined by measuring activity against casein and a fluorogenic substrate (MCA-Lys-Pro-Leu-Gly-Leu-DNP-Dpa-Ala-Arg-NH2, FS-6) (Sigma, USA) (Neumann et al., 2004). Casein and AMZ-tk were incubated at 55°C for 30 min in the presence of the indicated amount of the reagents. The activity to casein was determined using the spectrophotometric method described above or the proteolysis was detected by 12% SDS-PAGE. For proteolytic digestion of FS-6, 3.75 U of AMZ-tk and 0.5 μM FS-6 were incubated at 55°C for 5 min in the presence of the indicated amount of the reagents. The activity were monitored with a fluorescence spectrophotometer (Agilent, USA) with fluorescence excitation and emission at 328 and 393 nm, respectively.

SDS-PAGE method was used to detect self-cleavage of AMZ-tk. 15 U mL^-1^ of purified AMZ-tk was incubated with various metal ions and EDTA at 55°C for 30 min. The mixtures were detect by 12% SDS-PAGE and band density was further analyzed by ImageJ (Schneider et al., 2012).

For kinetic studies, the initial velocities of enzymatic reactions were examined by varying the concentration of casein (from 0.4 to 60 mg mL^-1^) under optimal conditions. The apparent Michaelis constant (*K_m_*) value and maximal velocity (*V_max_*) were obtained by mathematical calculations using Origin pro (8.0) software. All of the activity data were determined by three separate experiments with at least three technical replicates.

### Effect of Detergents on Stability of AMZ-tk

The suitability of the enzyme as a detergent additive was determined by examining its stability in the presence of 2% surfactants such as sodium dodecyl sulphate (SDS), Triton X-100, Tween 80 and some commercial laundry detergents (OMO, Liby, Ariel, Wheel, and Surf excel). The endogenous enzymes in the commercial detergents were inactivated by heating the diluted detergents for 30 min at 100°C prior to the addition of the AMZ-tk enzyme. AMZ-tk was incubated with these additives for 60 min at 37°C and then the residual enzyme activities were determined under the optimal assay conditions. Each experiment was tested in triplicates with at least three technical replicates.

### Antibacterial Activity of AMZ-tk

Antibacterial activity was measured by a liquid growth inhibition assay, performed in 96-wells microtiter plates (Tsuji et al., 1998). The purified AMZ-tk with final concentration 3.75 U mL^-1^ was added to 100 μL of a logarithmic phase culture of the tested bacteria which was diluted to 10^4^ colony-forming units (CFU) per mL in 300 mM NaCl, 0.8 mM ZnCl_2_, pH 7.5. Control wells were inoculated with the same volume of salt solution. Microbial growth was measured as an increase of optical density at 600 nm (OD_600_) by a microplate reader (MRX, Dynex, USA) after incubation at 25°C for 12 h. Antibacterial specificity of AMZ-tk was assayed by adding 10 μL of the protease with final concentrations in the range of 0.75–15 U mL^-1^. Minimal inhibitory concentrations (MIC) are expressed as intervals of concentrations: [a]–[b]; where [a] is the highest concentration tested at which the bacteria are growing and [b] being the lowest concentration causing 100% of growth inhibition (no change in OD_600_) (Rai and Mukherjee, 2009). Each experiment was tested in triplicates with at least three technical replicates.

### Quantitative Reverse Transcription-PCR (RT-qPCR)

Culture of *T. kodakarensis* KOD1were carried out in triplicate in 40 mL cultures in 50 mL serum bottles at 85°C anaerobically on a shaking incubator (150 rpm). The cells in the mid-log phase were shocked by exposure to 45, 55, 65, 75, 85, 95°C and incubating for 1 h (Jia et al., 2015). The cells were harvested and RNA was prepared with TRIzol reagent (Invitrogen, Carlsbad, CA, USA). To ensure complete removal of any contaminating DNA, all RNA preparations were given a DNAase treatment (Thermo Scientific Fermentas, Germany). RNA was quantified with a spectrophotometer and cDNA was synthesized with the Universal RiboClone cDNA Synthesis System (Promega, USA) according to the manufacturer’s protocols. The reaction products were serially diluted to find the adequate concentration for real-time PCR analysis using the following primers: 5′-GTA TCC GAA AGG GTT GAG GA-3′ and 5′-CCT TCT GGT GTT TCA AGG GT-3′. Real-time PCR was performed in CFX96 Real-Time PCR System ((Bio-Rad, USA), using SYBR Green PCR Master Mix (Toyobo, Japan). The relative fold changes were determined from cycle threshold (C_T_) values using the ΔΔC_T_ method. The reactions for detection of 16S rRNA (TKr05) levels were used for normalization between the different samples, which were amplified with primers: 5′-GTC CTG GCT GTA AAG GAT GC-3′ and 5′-CCG CCA ATT CCT TTA AGT TT-3′. The experiments were analyzed in three independent assays, with at least three technical replicates included in each PCR to ensure reproducibility.

### Bioinformatics Analysis

Multiple sequence alignments of protein sequences were performed using the ClustalW (version 2) program (Larkin et al., 2007). The unrooted phylogenetic trees were constructed with MEGA 5.0 using the Neighbour-Joining (NJ), Minimal Evolution (ME), and Maximum Parsimony (MP) methods and the bootstrap test carried out with 1000 iterations (Tamura et al., 2011). Protein–protein interactions were predicted using STRING set at high confidence (Franceschini et al., 2013), and Cytoscape was used for network visualization (Shannon et al., 2003). The protein function was predicted by BLAST (Altschul et al., 1997), SMART (Letunic et al., 2015), and I-TASSER (Zhang, 2008).

The three dimensional structure of AMZ-tk was modeled using the I-TASSER software (Zhang, 2008) using the crystal structure of archaemetzincin from *Methanopyrus Kandleri* as a template (PDB code: 2X7M). Zinc ions were docked to AMZ-tk by means of Autodock Vina version 1.1.2 (Trott and Olson, 2010). For Vina docking, the default parameters were used as described in the Autodock Vina manual. The top ranked pose as judged by the Vina docking score was subject to visually analyze using PyMOL 1.7 software (www.pymol.org).

### Statistical Analysis

All of the data are presented as mean and standard deviation (SD). Data analyses were carried out by analysis of variance (ANOVA) by using the software EXCEL. The ANOVA was used to determine statistically significant differences. If the probability was less than 0.05, namely *p* < 0.05.

## Results

### Bioinformatics Analysis of AMZ-tk

**FIGURE 1:**
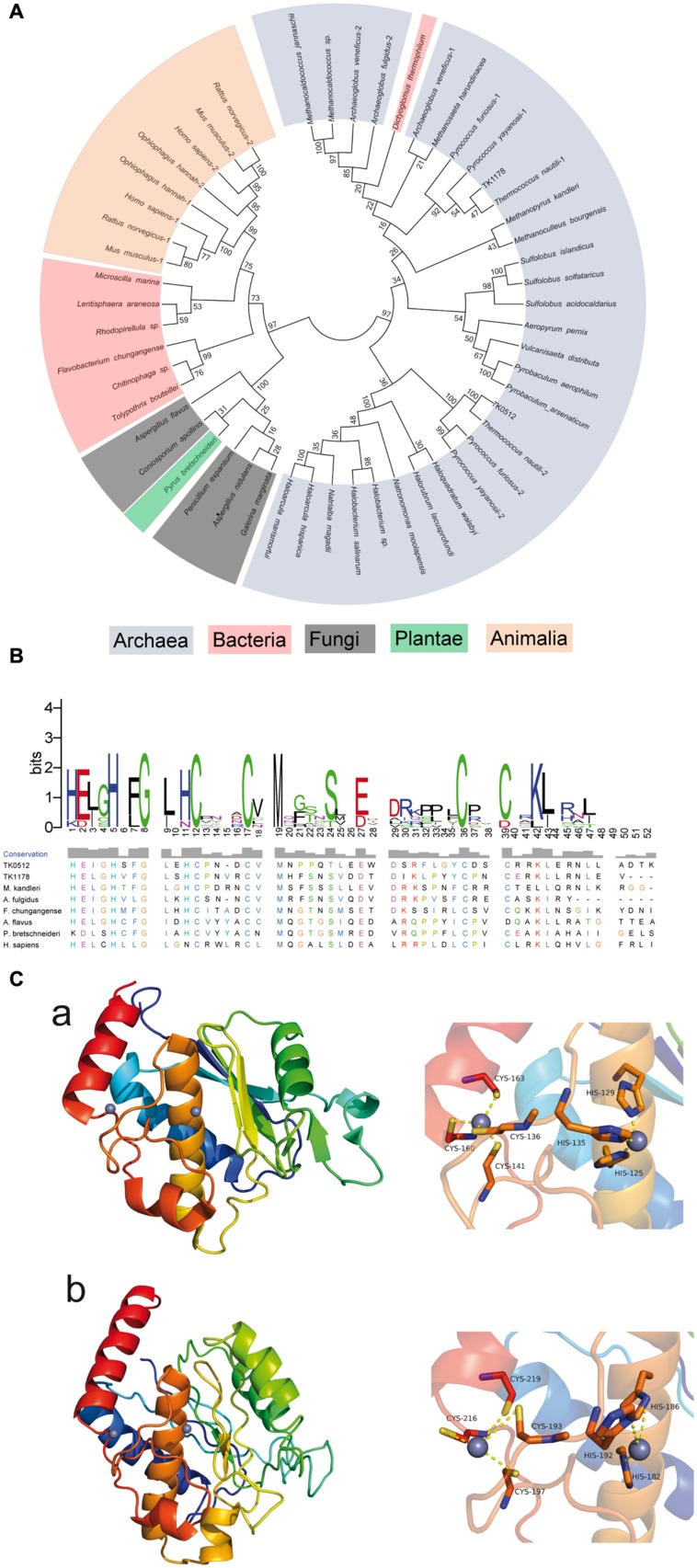
**Phylogenetic, sequence, and structure analysis of AMZ-tk and other archaemetzincins. (A)** Unrooted neighbor-joining phylogenetic tree for 50 archaemetzincins from archaea, bacteria, and eukarya (in different colors) generated using MEGA5. The optimal tree with a sum of branch length = 13.47550492 is shown. The percentage of replicate trees in which the associated taxa clustered together in the bootstrap test (1000 replicates) is shown next to the branches. **(B)** Sequence alignment of conserved zinc-binding motif of AMZ-tk and other homologs. The conserved zinc-binding motif of archaemetzincin from archaea (TK0512 (AMZ-tk), YP_182925.1; TK1178, YP_183591.1; *Methanopyrus kandleri*, WP_011018918.1; *Archaeoglobus fulgidus*, WP_010877837.1), bacteria (*Flavobacterium chungangense*, WP_031453746.1), fungi (*Aspergillus flavus*, XP_002379891.1), plantae (*Pyrus bretschneideri*, XP_009350639.1), and animalia (*Homo sapiens*, NP_001275985.1) was aligned by ClustalW. The conservation level of each residue is indicated by the height of the bars above each residue. The sequence logo was generated using WebLogo (http://weblogo.berkeley.edu/). **(C)** Homology modeling of AMZ-tk. The structure was generated by homology modeling using archaemetzincin from *M. Kandleri* (PDB code: 2X7M) as a template. (a) The overall structure (left) and Zinc-binding residues (right) in archaemetzincin from *M. Kandleri*. (b) The three-dimensional model structure (left) and Zinc-binding residues (right) in AMZ-tk.

AMZ-tk was annotated as a zinc-dependent protease in the original genome report (Fukui et al., 2005). A search of the sequence with NCBI BLAST-P revealed the most significant homology (64-35% identity) in the *Thermococcaceae* family of archaea, including *Palaeococcus*, *Pyrococcus*, and *Thermococcus* (Supplementary Figure S1). Following the proteins in the *Thermococcaceae* family, AMZ-tk is also similar to archaemetzincins in other organisms, such as the two archaemetzincins in humans and TK1178 in *T. kodakarensis* KOD1, which was annotated as archaemetzincin. To explore the evolutionary relationship of AMZ-tk and other annotated archaemetzincins, a phylogenetic tree was built by the MEGA5 program using amino acid sequence data for archaemetzincin from animals, plant, fungi, bacteria, and archaea (**Figure 1A**). The accession number of these proteins is listed in Supplementary Table S1 in the supplemental material. Although the bootstrap values were somewhat low due to the large number of sequences, more significant bootstrap values in the distal branches allowed us to infer that proteins from similar species are derived from a common ancestor, and AMZ-tk is closer to archaea than to bacteria and eukarya. Furthermore, protein positions in the dendrogram are independent of the methods for phylogenetic reconstruction (data not shown). These clustering results suggest that AMZ-tk is a new archaemetzincin that is closely related to some archaeal proteins.

To further verify that AMZ-tk is an archaemetzincin, sequence alignments by ClustalW were performed using the representative sequences from archaea, bacteria, and eukarya. The results show that AMZ-tk only shares 21.74% amino acid sequence identity to TK1178 in *T. kodakarensis* KOD1. The sequence identity to homologues from *M. kandleri, Archaeoglobus fulgidus*, *Penicillium expansum*, and *Homo sapiens* is also low (20.79, 18.75, 13.04, and 14.35%, respectively). However, AMZ-tk and other archaemetzincins possess the highly conserved HExxHxxGx_3_Cx_4_CxMx_17_CxxC motif with a conserved glutamate residue as the catalytic base, which are the main characteristics of archaemetzincin family proteins (**Figure 1B**). In the motif, HExxHxxGxxH with three histidine residues coordinate the first Zn^2+^ ion (Waltersperger et al., 2010). The second zinc ion is bound to four cysteines, thus resembling a zinc finger motif. There is also a conserved methionine that structurally supports the active site (Waltersperger et al., 2010; Graef et al., 2012).

To further confirm the presence of conserved structural motifs, the three-dimensional structure of the AMZ-tk was predicted by homology modeling methods using archaemetzincin from *M. Kandleri* (PDB code: 2X7M) as a template (Salamone et al., 2015). The predicted three-dimensional structure (**Figure 1C**) revealed C-terminus of AMZ-tk for Zn^2+^ ions binding is more highly conserved in structure. And the N-terminus of AMZ-tk prefer to form loop structure. Residues binding Zn^2+^ ions are absolutely conserved. In the structure of archaemetzincin from *M. Kandleri*, the first Zn^2+^ ion is chelated with residues His125, His129, and His135, while another chelation is observed between Zn^2+^ and residues Cys136, Cys141, Cys160, and Cys163. In AMZ-tk, chelation is observed between the first Zn^2+^ and residues His182, His186, and His192, whereas the second Zn^2+^ is chelated with residues Cys193, Cys197, Cys216, and Cys219.

### Expression and Purification of AMZ-tk from *E. coli*

**FIGURE 2:**
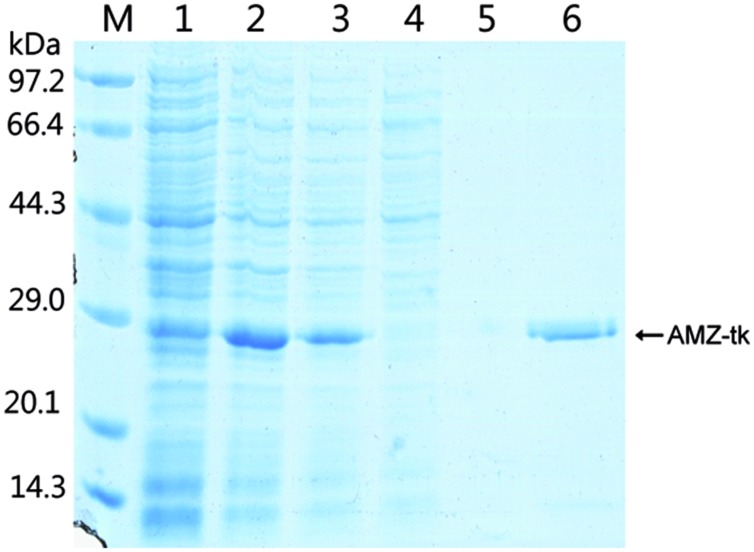
**Expression and purification of AMZ-tk from *Escherichia coli*.** Lane M, protein marker; the molecular mass standards are indicated at the left. Lane 1, crude protein extract from non-induced cells; Lane 2, crude protein extract from IPTG-induced cells; Lane 3, soluble extract from IPTG-induced cells after heating at 70°C for 1 h; Lane 4, unbound proteins eluted from the Ni-NTA column; Lane 5, proteins eluted with 20 mM imidazole; Lane 6, proteins eluted with 300 mM imidazole.

To study the function of the AMZ-tk, we first expressed the gene by IPTG induction in a bacterial system (**Figure 2**). As indicated in lane 3, a protein band at approximately 28 kDa was detected after IPTG induction, which matched with the predicted molecular mass (27.6 kDa) of AMZ-tk. After cultivation, *E. coli* cells were resuspended in the required buffer and sonicated. After centrifugation, recombinant AMZ-tk was effectively purified by Ni-NTA affinity chromatography, which produced a single band in the SDS–PAGE gel (**Figure 2**).

### Enzymatic Characterization of AMZ-tk

**FIGURE 3:**
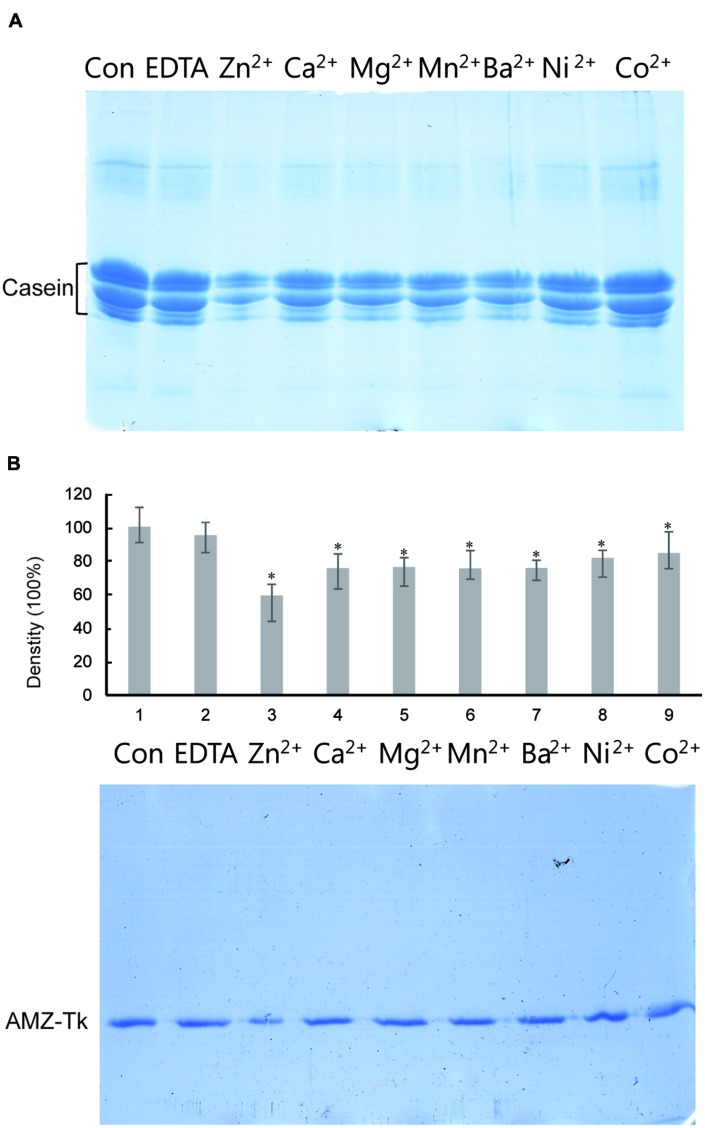
**Degradation of casein by AMZ-tk and self-cleavage activity of purified AMZ-tk analyzed by SDS-PAGE. (A)** Degradation of casein by AMZ-tk. Purified AMZ-tk was incubated with casein at 55°C in the presence of 0.08 mM divalent ions or 5 mM EDTA for 30 min. Casein alone was used as control. Samples were analyzed using 12% SDS-PAGE and Coomassie Brilliant Blue staining. **(B)** Self-cleavage of purified AMZ-tk. Purified AMZ-tk was incubated at 55°C in the presence of 0.08 mM divalent ions or 5 mM EDTA for 30 min. Fresh purified protein was used as control. Samples were analyzed using 12% SDS-PAGE and Coomassie Brilliant Blue staining. The upper panel shows the density of protein bands. ^∗^*p* < 0.05 compared with fresh purified protein.

The sequence analysis results suggested that AMZ-tk was an archaemetzincin. A series of enzyme activity assays were performed to elucidate its characteristics. Firstly, the activity was assayed in the presence of different divalent ions, which is one of the many external factors that affect the catalytic activity of these enzymes (Shivanand and Jayaraman, 2011). AMZ-tk had proteolytic activity in the presence of Zn^2+^, Ca^2+^, Mg^2+^, Mn^2+^, Ba^2+^, and Ni^2+^ using casein-based spectrophotometric assays; the addition of Zn^2+^ induced the highest activity. EDTA, a metal chelator, caused a significant decrease in the activity. Similar result was shown by using fluorescent peptide as a substrate (**Table 1**). We next examined the ability of AMZ-tk to proteolyse casein using SDS-PAGE (**Figure 3A**). When casein was incubated with AMZ-tk in the presence of Zn^2+^, its concentration decreased dramatically. A similar decrease was also observed in the presence of other divalent ions. These results suggest that the purified protease is a metal protease, which is in agreement with the sequence analysis. Interestingly, no degradation products were visible in SDS-PAGE, even BSA was used as the substrate (data not shown). We propose that maybe the products are too small to be detected in SDS-PAGE. Meanwhile, we consistently observed a marked reduction in the amount of AMZ-tk irrespective of the presence or absence of casein. The addition of the chelator was essential to prevent self-degradation of AMZ-tk (**Figure 3B**). In addition, self-degradation was not detected if the reaction temperature is lower than 40°C (data not shown). Thus, AMZ-tk not only is able to degrade other substrate proteins but also shows self-cleavage activity.

**Table 1:** Effects of various metal ions and protease inhibitors (with 0.8 mM ZnCl_2_) on the activity of AMZ-tk.

Factors	Concentration	Relative activity to casein (%)	Relative activity to FS-6 (%)
Zn^2+^	0.8 mM	100	100
Ca^2+^	0.8 mM	55.9 ± 1.14^∗^	65.2 ± 5.68^∗^
Mg^2+^	0.8 mM	69.6 ± 1.72^∗^	63.8 ± 4.37^∗^
Mn^2+^	0.8 mM	66.0 ± 2.42^∗^	58.6 ± 6.32^∗^
Ba^2+^	0.8 mM	64.0 ± 4.45^∗^	70.1 ± 3.58^∗^
Ni^2+^	0.8 mM	55.9 ± 1.25^∗^	50.3 ± 6.25^∗^
Co^2+^	0.8 mM	36.1 ± 3.20^∗∗^	32.1 ± 4.47^∗∗^
EDTA	2.0 mM	5.9 ± 0.24^∗∗^	3.2 ± 1.25^∗∗^
EGTA	2.0 mM	6.1 ± 0.46^∗∗^	4.5 ± 0.54^∗∗^
PMSF	2.0 mM	92.7 ± 5.18	90.1 ± 8.10
DTNB	2.0 mM	87.0 ± 2.86^∗^	93.5 ± 6.83

*Casein and FS-6 peptide was used as substrates to measure the activity of AMZ-tk as described under Section ‘Materials and Methods.’ The activity in the presence of 0.8 mM Zn^2+^ was set 100%. ^∗^p < 0.05 and ^∗∗^p < 0.01 compared with control.*

**FIGURE 4:**
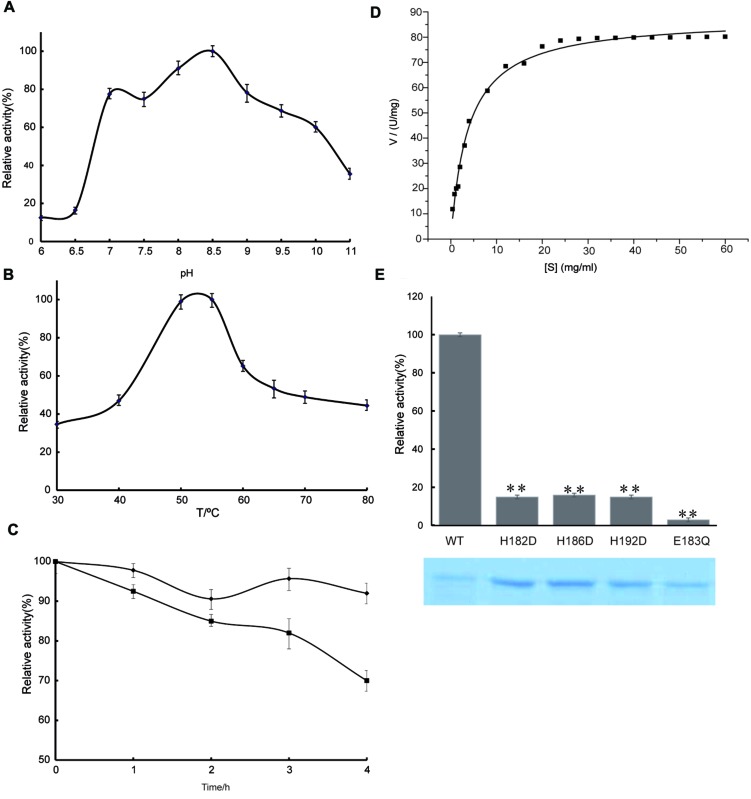
**Enzyme activity assay of AMZ-tk. (A)** Optimal pH of AMZ-tk. Different buffers were used for the different pH solutions in this assay. MES buffer was used for pH 5.0–7.5; HEPES buffer was used for pH 8 and 8.5; glycine buffer was used for pH 9.0 and 10.0; and Na_2_HPO_4_ buffer was used for pH 11.0. Values obtained at pH 8.5 were set as 100%. **(B)** Optimal temperature of AMZ-tk. Values obtained at 55°C were set as 100%. **(C)** After inactivation of AMZ-tk at 70°C (filled diamonds) and at 80°C (filled squares) for 1–4 h, the activity of the enzyme was assayed at 55°C as described in Section “Materials and Methods.” **(D)** Effects of substrate concentration on the velocity of the AMZ-tk. Assays were performed as described in Section “Materials and Methods.” **(E)** Relative activity of wild-type AMZ-tk, H182D, H186D, H192D and E183Q with casein as substrate. The lower part shows the corresponding proteins determined by SDS-PAGE. The parameters reported here are the means of three determinations. ^∗∗^*p* < 0.01 compared with wild-type AMZ-tk.

To study the effect of pH, the protease activities of the purified enzyme were surveyed between pH values of 6.0 and 11.0 using casein as a substrate at an assay temperature of 55°C. The pH profile of the purified AMZ-tk is shown in **Figure 4A**. The enzyme was most active between pH values 7.0–10.0, with maximal activity at pH 8.0–9.0. Protease activity was approximately 50% of the maximal activity when assayed at pH 10.0 and decreased significantly below pH 7.0. The remarkable activity with a pH higher than 7.0 reveals the highly alkaline nature of this protease, which makes it suitable for applications in alkaline environments.

In the temperature activity experiments, the protease activities of AMZ-tk were measured at temperatures ranging from 30°C to 80°C at a constant pH of 7.5. The results revealed that the optimum temperature for the enzyme was 55°C (**Figure 4B**). Under the optimal catalytic conditions, AMZ-tk can degrade casein with a specific activity of 74.7 U mg^-1^ (*n* = 12). A comparison of the catalytic properties of AMZ-tk and thermophilic proteases from other organisms is shown in Supplementary Table S2.

The thermostability of the protease was examined by measuring the remaining activities at 55°C after incubation of the enzyme without Zn^2+^ at 70 and 80°C for 1–4 h. At 70°C, the enzyme retained almost full activity after 4 h of incubation. At 80°C, 30% of the original activities were lost after 4 h of incubation (**Figure 4C**). These results suggest that AMZ-tk is thermostable, which is one of the good characteristics of the protease.

The kinetics of recombinant AMZ-tk were analyzed using casein as a substrate by varying its concentration. The Michaelis–Menten equation was used to calculate the kinetic parameters (**Figure 4D**). AMZ-tk catalyzed casein with an apparent *K_m_* = 4.4 ± 0.2 mg mL^-1^ and *V_max_* = 90.4 ± 3.2 U mg^-1^ (*n* = 15).

To verify the function of predicted active amino acid residues in sequence analysis, three point mutants at Zinc binding site (H182D, H186D, and H192D) and one mutant at catalytic base (E183Q) was purified, using the same methods as for the wild-type AMZ-tk (**Figure 4E**). The hydrolyzing activity toward casein by the point mutants at Zinc binding site (H182D, H186D, and H192D) was approximately 20% of that of wild-type AMZ-tk. The detectable activity for the single amino acid mutant may be caused by that the mutant could still bind Zn^2+^ but not as efficiently as the wild-type protein. The E183Q mutant exhibited minimal enzymatic activity, indicating that the zinc binding and catalytic base are all important residues in AMZ-tk.

In order to determine the nature of AMZ-tk, the effect of a variety of enzyme inhibitors, such as chelating agent and a specific group reagent on enzyme activity was investigated (**Table 1**). The cysteine protease inhibitor [5, 5-Dithiobis (2-nitrobenzoic acid), DTNB] and serine protease inhibitor (Phenylmethylsulfonyl fluoride, PMSF) were without influence on the activity of the purified enzyme. However, the enzyme was completely inhibited by the metalloenzyme inhibitor EDTA and EGTA, indicating that the enzymes belonged to the metalloprotease family.

### Detergents Compatibility of AMZ-tk

To check the compatibility and stability of AMZ-tk towards detergents, enzymes were pre-incubated in the presence of various laboratory or commercial laundry detergents at 37°C for 1 h (**Table 2**). It is notable that AMZ-tk retained >50% activity in the presence of 2% laboratory surfactants (SDS and Triton). Moreover, AMZ-tk exhibited higher stability in commercial laundry detergents. The enzyme retained 88% of its initial activity with Ariel and followed by Wheel, OMO, Liby, Surf excel, and Tide (70–65%). The obtained results clearly indicated that AMZ-tk is suitable for various industrial applications based on the performance of enzymes in detergents.

**Table 2:** Effects of various detergents on the activity of AMZ-tk.

Detergents	Concentration	Relative activity (%)
SDS	2%	56.1 ± 0.56
Triton	2%	52.7 ± 0.78
Tween	2%	60.7 ± 6.58
Ariel	2%	88.3 ± 5.68
Wheel	2%	79.5 ± 5.52
Surf excel	2%	72.0 ± 6.48
OMO	2%	75.6 ± 5.24
Liby	2%	73.5 ± 8.61
Tide	2%	65.9 ± 6.15

*The enzymes were pre-incubated with detergents for 60 min at 37°C and the remaining protease activities were measured under standard conditions. The activity is expressed as a percentage of the activity level in the absence of additives. Data were statically analyzed by one-way ANOVA (p < 0.05).*

### Antibacterial Activity of AMZ-tk

We examined the antibacterial activity and specificity of the AMZ-tk protease by assessing inhibition of the growth of various microorganisms (**Table 3**). The protease inhibited the growth of both Gram-negative bacteria (*E. coli*, *Yersinia enterocolitica*, and *Klebesilla pneumoniae*) and Gram-positive bacteria (*Staphylococcus aureus*, *Bacillus cereus*, and *Listeria monocytogenes*) as summarized in **Table 3**. And AMZ-tk showed the significantly higher inhibition of growth of the Gram-positive bacteria than that of the Gram-negative bacteria. The minimum inhibitory concentration (MIC) value to Gram-negative bacteria was also higher than that to Gram-positive bacteria under our assay conditions, suggesting that Gram-positive bacteria were very sensitive to this enzyme.

**Table 3:** Antimicrobial activity and specificity of the AMZ-tk protease.

Microorganisms	Growth inhibition (%)	MIC value (U mL^-1^)
*Escherichia coli*	22 ± 3.5	11.5 ± 0.8
*Yersinia enterocolitica*	32 ± 5.2	9.2 ± 0.6
*Klebesilla pneumoniae*	37 ± 4.8	8.5 ± 1.1
*Staphylococcus aureus*	74 ± 9.2	3.1 ± 0.4
*Bacillus cereus*	66 ± 8.7	3.9 ± 0.4
*Listeria monocytogenes*	85 ± 10.6	2.1 ± 0.3

*Growth inhibition and minimal inhibitory concentration (MIC) of AMZ-tk against various Gram-negative and Gram-positive bacterial strains were measured by incubation of the enzyme with the strains. The growth of the strains was recorded by spectrophotometric methods and the values were calculated as described under Section ‘Materials and Methods.’*

### Expression of *AMZ-tk* Gene

**FIGURE 5:**
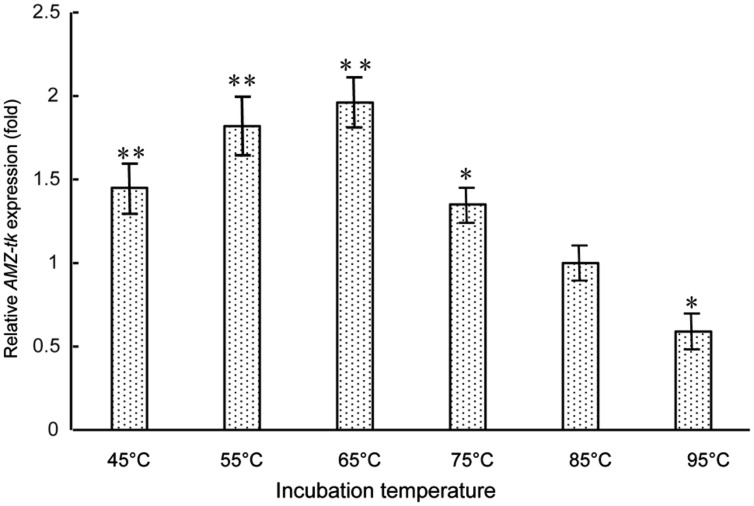
**Expression level of *AMZ-tk* gene at different temperatures.** The mRNA relative quantity of *AMZ-tk* from *T. kodakaraensis* KOD1 cells treated by different temperatures was measured by RT-qPCR and indicated as fold difference from the value of the cells growing at 85°C, which is taken as 1. Error bars indicate the standard deviations from three independent experiments. ^∗^*p* < 0.05 and ^∗∗^*p* < 0.01 compared with the expression level of *AMZ-tk* at 85°C.

As AMZ-tk showed optimal temperature at 55°C, which is lower than the optimal growth temperature of *T. kodakarensis* KOD1 (85°C), gene expression level was studied by RT-PCR to investigate the physiological role of AMZ-tk. RNA was isolated from *T. kodakarensis* KOD1 cells exposed to 45–95°C. The relative expression patterns of *AMZ-tk* at different temperature (45–95°C) are shown in **Figure 5**. The lowest transcription level could be seen at 95°C, which is higher than the optimal growth temperature. With temperature decreased, the expression level increased and reached the highest transcription level at 65°C. The transcription level then declined slightly at 45°C and 55°C. Therefore, the physiological function of *AMZ-tk* seemed to be related to cold adaptation.

## Discussion

Sequence analysis of AMZ-tk suggested that the protein contains an archaemetzincin active site and is a metal protease. However, no direct evidence of its proteolytic activity was available. In this work, we purified and characterized AMZ-tk to address this issue. The purified protease was capable of degrading casein and exhibited self-cleavage activity. These reactions were dependent on the presence of zinc and inhibited by EDTA. These results indicated that AMZ-tk is a zinc metalloprotease. The activity of the purified enzyme was higher in alkaline pH. AMZ-tk was thermostable at 80°C, although its optimal catalytic temperature was 55°C.

While our understanding of the catalytic mechanisms of archaemetzincin is improving, many of its physiological roles remain unknown (Waltersperger et al., 2010). AMZ-tk showed optimal catalytic temperature at 55°C, lower than the optimal growth temperature of the cell. Some proteins in thermophilic archaea also showed low temperature optimal catalytic characteristics, such as cyclodextrinase from *T. kodakarensis* (Sun et al., 2015). Furthermore, the transcription level of *AMZ-tk* also increased at low temperature. Using these findings, we propose that AMZ-tk may have function under “cold” conditions. On the other hand, the two archaemetzincins in *T. kodakarensis* KOD1 (AMZ-tk and TK1178) contain the same zinc-binding motif, protein–protein interaction prediction (score >80%) indicates that AMZ-tk and TK1178 may interact with different proteins (Supplementary Figure S2). AMZ-tk may interact with detoxifying proteins while TK1178 may regulate DNA metabolism. Both of them may interact with *N*-acetyltransferase and leucyl-tRNA synthetase. Thus, AMZ-tk and TK1178 might have different physiological roles.

The evolution of archaemetzincins is also complex. For example, archaemetzincin from plant is in the same cluster of proteins from fungi and archaemetzincin from thermophilic bacteria (*Dictyoglomus thermophilum*) is more closely related to the proteins from archaea (**Figure 1**). In *T. kodakarensis* KOD1 and other *Thermococcaceae* there are two archaemetzincins, which is explained by gene duplication (Diaz-Perales et al., 2005). However, gene duplications in the *T. kodakaraensis* genome are rare, and the duplicated genes are always of high identity. For example, TK0298, TK0495, and TK0850 are 87–90% identical at the amino acid level (Fukui et al., 2005). However, AMZ-tk and TK1178 showed only 20% sequence identity. From phylogenetic analysis, AMZ-tk is more closely related to the proteins from the class of *Haloarchaea*, and TK1178 is in the cluster of proteins from *Archaeoglobi*. Based on the sequence analysis, the occurrence of AMZ-tk in *T. kodakarensis* KOD1 is expected to have arisen not only from gene duplication but also from lateral gene transfer.

Many industrial microbial proteases are metalloproteases, such as Thermoase PC10F (Amano Enzyme Inc., Japan) and Neutrase (Novo Nordisk, Denmark), which are widely used in the food processing, medicine, brewing, leather, film, and baking industries (Wu and Chen, 2011). A number of thermostable metalloproteases from *Vibrio*, *Sulfolobus*, *Bacillus*, and *Thermomicrobium* have also been reported (Murao et al., 1991; Colombo et al., 1992; Fukuda et al., 1998; Aqel et al., 2012). Comparisons of these enzymes are shown in Supplementary Table S2. Among them, the protease from *Bacillus* is the only neutral protease; the others are all alkaline proteases. Owing to the better cleansing properties of protease over conventional synthetic detergents, alkaline proteases have made their way as key-ingredients in detergent formulations (Rai and Mukherjee, 2009). Recently, some proteases have been reported to have potential application as a laundry detergent additive (Venugopal and Saramma, 2007; Jaouadi et al., 2008; Manni et al., 2010; Jain et al., 2012). AMZ-tk also showed valuable feathers as an additive in laundry detergent. Interestingly, AMZ-tk inhibited the growth of several pathogenic organisms, suggesting that AMZ-tk has many applications in the food industry replacing other proteases to control the presence of pathogenic bacteria and to extend the shelf life of fresh and processed food. Although AMZ-tk has self-degradation activity, the reaction only can only be observed above 40°C. Due to the advantages mentioned above (thermostability, detergent-tolerant and antibacterial activity), AMZ-tk may also be a potential biocatalyst for industrial applications.

## Author Contributions

Conceived and designed the experiments: BJ and CJ. Performed the experiments: BJ, ZL, and JL. Contributed reagents/materials/analysis tools: YS, XJ, YX, and JZ. Wrote the paper: BJ.

## Conflict of Interest Statement

The authors declare that the research was conducted in the absence of any commercial or financial relationships that could be construed as a potential conflict of interest.
