# Supplementary material for: A Zinc-Dependent Protease AMZ-tk from a Thermophilic Archaeon is a New Member of the Archaemetzincin Protein Family
Source: Front Microbiol. 2015 Dec 17;6:1380. doi: 10.3389/fmicb.2015.01380 (PMC4681839; doi:10.3389/fmicb.2015.01380)
Supplement: Supplementary file 1 [file Data_Sheet_1.PDF]

A zinc-dependent protease AMZ-tk from a thermophilic archaeon is a new member of the archaemetzincin protein family

Supplementary Fig. 1

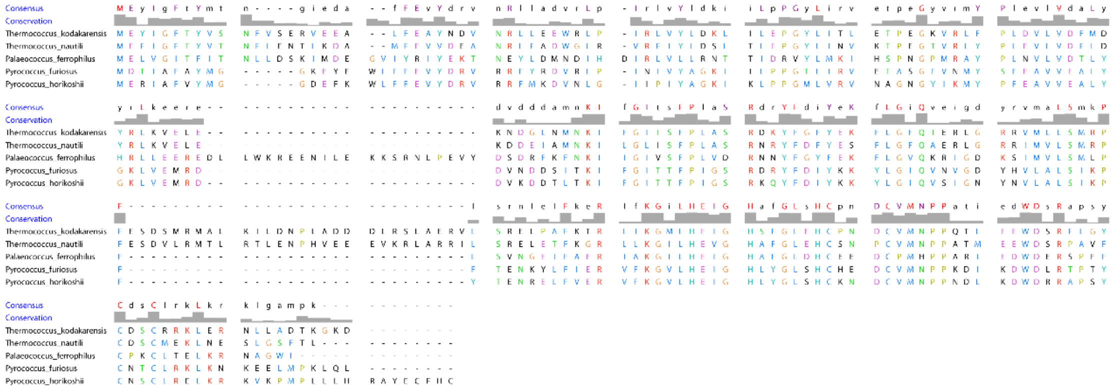

Supplementary Fig. 1 Sequence analysis of AMZ-tk. Archaemetzincin sequences from *Thermococcus kodakarensis* (YP_182925.1), *Thermococcus nautili* (WP_042689681.1), *Pyrococcus_furiosus*(WP_011012973.1), *Pyrococcus horikoshii* (WP_010885849.1), and *Palaeococcus_ferrophilus* (WP_048151914.1) were aligned. The conservation level of each residue is indicated by the height of the bars above each residue.

Supplementary Fig. 2.

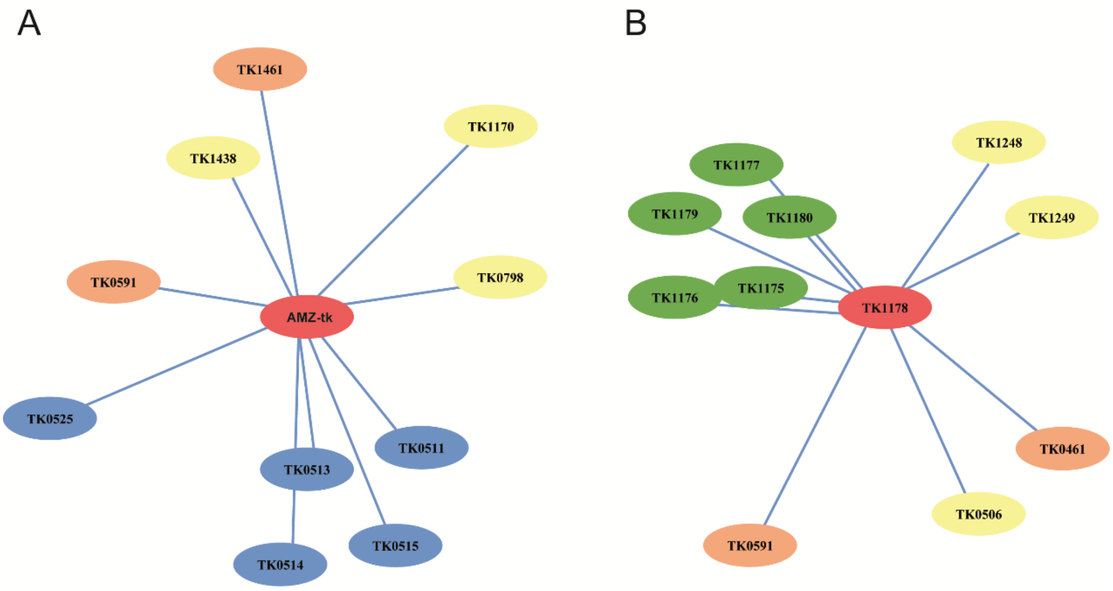

Supplementary Fig. 2. Predicted protein-protein interaction network of AMZ-tk (A) and TK1178 (B). The graph was constructed in the STRING tools using standard parameters. The proteins that may be involved in detoxifying are shown in blue and the proteins that may regulate DNA metabolism are shown in green. The proteins that may interact with both AMZ-tk and TK1178 are shown in orange. These proteins are annotated as: TK0506, translation-associated GTPase; TK0511, pyruvate kinase; TK0514, camphor resistance protein CrcB; TK0525, superoxide reductase; TK0591, N-acetyltransferase; TK0798, DNA topoisomerase VI subunit A; TK1175, type Holliday junction resolvase; TK1177, deblocking aminopeptidase; TK1179, adenylate cyclase; TK1438, fibronectin-binding protein; TK1461, leucyl-tRNA synthetase; TK1770, cyclomaltodextrinase; and TK0513, TK0515, TK1180, TK1176, TK1248, and TK1249 are hypothetical proteins. Protein functions were predicted using the software listed in ‘Materials and Methods.’

Supplementary Table I. Archaemetzincin sequences from different organisms used for phylogenetic analysis.

Organism	Gene Bank Accession No.
Aeropyrum pernix	WP_010865524.1
Archaeoglobus fulgidus	WP_010877837.1
***Archaeoglobus veneficus*-1**	YP_004342225.1
***Archaeoglobus veneficus*-2**	WP_013682907.1
Aspergillus flavus	XP_002379891.1
Aspergillus nidulans	CBF83223.1
Chitinophaga sp.	WP_029464671.1
Coniosporium apollinis	XP_007781354.1
Dictyoglomus thermophilum	WP_012547438.1
Flavobacterium chungangense	WP_031453746.1
Galerina marginata	KDR81884.1
Haloarcula hispanica	YP_008877092.1
Haloarcula marismortui	YP_136662.2
Halobacterium salinarum	WP_010903951.1
***Halobacterium* sp.**	AHG02721.1
Haloquadratum walsbyi	WP_021055743.1
Halorubrum lacusprofundi	YP_002565008.1
***Homo sapiens*-1**	NP_597720.1
***Homo sapiens*-2**	NP_001275985.1
Lentisphaera araneosa	WP_007280897.1
Methanocaldococcus jannaschii	WP_010869779.1
Methanocaldococcus sp.	WP_012979875.1
Methanoculleus bourgensis	YP_006545080.1
Methanosaeta harundinacea	WP_014586234.1
Microscilla marina	WP_002697137.1
***Mus musculus*-1**	NP_775581.1
***Mus musculus*-2**	NP_001239122.1
Natrialba magadii	YP_003479690.1
Natronomonas moolapensis	WP_015408079.1
***Ophiophagus hannah*-1**	ETE59834.1
***Ophiophagus hannah*-2**	ETE71868.1
Penicillium expansum	KGO44057.1
Pyrobaculum aerophilum	WP_011008336.1
Pyrobaculum arsenaticum	WP_011901510.1
***Pyrococcus furiosus*-1**	WP_011013000.1
***Pyrococcus furiosus*-2**	WP_011012973.1
***Pyrococcus yayanosii*-1**	WP_013904868.1
***Pyrococcus yayanosii*-2**	*WP_013904896.1*
Pyrus bretschneideri	*XP_009350639.1*
***Rattus norvegicus*-1**	NP_001040557.1
***Rattus norvegicus*-2**	NP_001014143.1
Rhodopirellula sp.	WP_009102142.1
Sulfolobus acidocaldarius	WP_011277458.1
Sulfolobus islandicus	WP_012711449.1
Sulfolobus solfataricus	WP_009991235.1
**TK0512**	YP_182925.1
**TK1178**	YP_183591
Tolypothrix bouteillei	WP_038111967.1
Vulcanisaeta distributa	YP_003901466.1

Supplementary Table II. Comparison of the biochemical properties of protease-Tk and other proteases.

	MW (KDa)	Optimal pH	Optimal temp	Specific activity (U/mg)	References
AMZ-tk	28	8.0-9.0	55 ℃	78.37	This study
Protease-Vs	48	9.5-10.0	55 ℃	467	Fukuda et al., 1998
Protease-Ts	35	8.5	75 ℃	N.D.	Murao et al., 1991
Protease-Ss	42	7.0	70 ℃	5.65	Colombo et al., 1992
Protease-Bs	48	6.8	80 ℃	744	Aqel et al., 2012

The proteases are from *T. kodakarensis* KOD1 (AMZ-tk), Vibrio sp. NUF-BPP-1 (Protease-Vs), *Sulfolobus solfatavicus (*Protease-Ss*), Bacillus* strain HUTBS62 (Protease-Bs), and *Thermomicrobium* sp. KN-22 (Protease-Ts). N.D.: not determined.

References:

Aqel, H., F.Al-Quadan., and Yousef T.K. (2012). A novel neutral protease from thermophilic *Bacillus* strain HUTBS62. J. BioSci. Biotech. 1, 117-123.

Colombo, S., D'auria, S., Fusi, P., Zecca, L., Raia, C.A., and Tortora, P. (1992). Purification and characterization of a thermostable carboxypeptidase from the extreme thermophilic archaebacterium *Sulfolobus solfataricus*. Eur. J. Biochem. 206, 349-357. doi: 10.1111/j.1432-1033.1992.tb16934.x

Fukuda, K., Hamaguchi, N., Oda, T., Ishimatsu, A., and Muramatsu, T. (1998). Hemagglutinating activity of extracellular alkaline metalloendopeptidases from *Vibrio* sp. NUF-BPP1. Biosci. Biotechnol. Biochem. 62, 1157-1160.doi:10.1271/bbb.62.1157

Murao, S., Nomura, Y., Nagamatsu, K., Hirayama, K., Iwahara, M., and Shin, T. (1991). Purification and some properties of a thermostable metal proteinase produced by *Thermomicrobium* sp. KN-22 strain. Agric. Biol. Chem. 55, 1739-1744.doi:10.1271/bbb1961.55.1739

Colombo, S., D'auria, S., Fusi, P., Zecca, L., Raia, C.A., and Tortora, P. (1992). Purification and characterization of a thermostable carboxypeptidase from the extreme thermophilic archaebacterium Sulfolobus solfataricus. Eur. J. Biochem. 206, 349-357. doi: 10.1111/j.1432-1033.1992.tb16934.x
